# Anti-tumor treatment and healthcare consumption near death in the era of novel treatment options for patients with melanoma brain metastases

**DOI:** 10.1186/s12885-022-09316-7

**Published:** 2022-03-05

**Authors:** Annemarie C. Eggen, Geke A. P. Hospers, Ingeborg Bosma, Miranda C. A. Kramer, Anna K. L. Reyners, Mathilde Jalving

**Affiliations:** 1 Department of Medical Oncology, University of Groningen, University Medical Center Groningen, Groningen, the Netherlands; 2 Expertise Centre Palliative Care, University of Groningen, University Medical Center Groningen, Groningen, the Netherlands; 3 Department of Neurology, University of Groningen, University Medical Center Groningen, Groningen, the Netherlands; 4 Department of Radiation Oncology, University of Groningen, University Medical Center Groningen, Groningen, the Netherlands

**Keywords:** Healthcare consumption, End-of-life care, Anti-tumor treatment, Melanoma, Neuro-oncology

## Abstract

**Background:**

Effective systemic treatments have revolutionized the management of patients with metastatic melanoma, including those with brain metastases. The extent to which these treatments influence disease trajectories close to death is unknown. Therefore, this study aimed to gain insight into provided treatments and healthcare consumption during the last 3 months of life in patients with melanoma brain metastases.

**Methods:**

Retrospective, single-center study, including consecutive patients with melanoma brain metastases diagnosed between June-2015 and June-2018, referred to the medical oncologist, and died before November-2019. Patient and tumor characteristics, anti-tumor treatments, healthcare consumption, presence of neurological symptoms, and do-not-resuscitate status were extracted from medical charts.

**Results:**

100 patients were included. A *BRAF-*mutation was present in 66 patients. Systemic anti-tumor therapy was given to 72% of patients during the last 3 months of life, 34% in the last month, and 6% in the last week. Patients with a *BRAF-*mutation more frequently received systemic treatment during the last 3 (85% vs. 47%) and last month (42% vs. 18%) of life than patients without a *BRAF-*mutation. Furthermore, patients receiving systemic treatment were more likely to visit the emergency room (ER, 75% vs. 36%) and be hospitalized (75% vs. 36%) than those who did not.

**Conclusion:**

The majority of patients with melanoma brain metastases received anti-tumor treatment during the last 3 months of life. ER visits and hospitalizations occurred more often in patients on anti-tumor treatment. Further research is warranted to examine the impact of anti-tumor treatments close to death on symptom burden and care satisfaction.

## Background

Up to 50% of the patients with metastatic melanoma eventually develop brain metastases, which are associated with increased morbidity and mortality [1–3]. Local treatments for brain metastases (whole-brain radiation (WBRT), stereotactic radiotherapy (SRT), and neurosurgery) have long been used to temporarily decrease symptoms associated with melanoma brain metastases. Next to these local treatments, patients with metastatic melanoma could also be treated with various systemic anti-tumor treatments (e.g., various chemotherapeutic agents and interleukin-2). However, these older systemic treatments lacked intracranial efficacy. Historically, the survival from diagnosis of brain metastases was limited to 4 to 5 months [4–6]. The introduction of effective systemic therapies between 2011 and 2015 revolutionized the management of melanoma brain metastases and, depending on prognostic factors, the median survival from brain metastases diagnosis currently ranges from 5 to 34 months [7–12]. The availability of these novel systemic treatments has resulted in more patients receiving anti-tumor treatment. However, not all patients will benefit from these treatments and most patients will still die of their disease. Currently, it is unknown to which extent the effective treatment options influence disease trajectories close to death in patients with melanoma brain metastases.

The treatment options that revolutionized melanoma management are immune checkpoint inhibitors and targeted therapies. Intracranial responses to immune checkpoint inhibitors are observed, and these responses can be durable [9, 13–16]. The highest response rates are reported in patients with asymptomatic brain metastases, and these range from 46 to 57% [15–17]. In patients with leptomeningeal disease, neurological symptoms, or patients that progressed on localized treatment, response rates are lower (5 to 22%) [15–17]. For patients harboring a *BRAF-*mutation, approximately 50 to 60% of patients with cutaneous melanoma, BRAF/MEK-inhibitors are also available [18, 19]. Intracranial responses with BRAF/MEK-inhibitors are independent of the presence of symptoms and range from 44 to 68% [20, 21]. Furthermore, the onset of response and relief of symptoms with BRAF/MEK-inhibitors is often within days and more rapid than for immune checkpoint inhibitors. Unfortunately, the duration of intracranial responses to BRAF/MEK-inhibitors are limited (4 to 6 months) [21–23]. BRAF/MEK-inhibitors can be initiated to induce rapid tumor response and provide symptom relief and to create the opportunity to commence other treatments, including immune checkpoint inhibitors, at a later time point. Re-challenge with BRAF/MEK-inhibition and continuation of BRAF/MEK-inhibitors post-progression to prevent rapid intracranial progression have both been reported to provide clinical benefit [24–27]. These can be a reason to continue or recommence BRAF/MEK-inhibitors even in the end-of-life phase.

Studies examining end-of-life care in cancer patients often assess provided treatments near death, emergency department (ER) visits, hospitalizations, and the number of patients dying at the preferred place of death [28–31]. However, most of such studies in melanoma were performed before the implementation of effective systemic treatments [32–38]. These treatments have significantly changed the management of melanoma brain metastases, including end-of-life care. Because of the ongoing possibility of long-term survival with immune checkpoint inhibitors and the palliative effect of BRAF/MEK-inhibitors, high numbers of patients may be receiving anti-tumor treatment near death. The current study was performed to obtain insight into the anti-tumor treatment and hospital healthcare consumption during the last 3 months of life in patients with melanoma brain metastases. Furthermore, this study may identify current knowledge gaps in the end-of-life care of patients with melanoma brain metastases.

## Methods

### Study design and patient selection

This retrospective, single-center cohort study included all melanoma patients diagnosed with brain metastases between June-2015 and June-2018, referred to the Department of Medical Oncology of the University Medical Center Groningen (UMCG), the Netherlands, and died before November-2019. Data was collected from June-2015 because the currently used effective treatments were implemented as standard of care at that time. The UMCG ethical review board granted ethical approval and waived the need for an informed consent procedure (METc2017/511). The “opt-out” register was assessed to exclude patients who disapproved of routinely collected data used for research purposes.

### Healthcare in the Netherlands and the UMCG

In the Netherlands, all inhabitants have access to a general practitioner (GP), and healthcare at home can be provided for those in need. Costs associated with the needed long-term nursing and/or 24-h healthcare are paid from tax incomes. Subsequently, most terminally ill cancer patients can continue to live at home during the last phase of life [39]. The Dutch government considers palliative care to be the responsibility of all healthcare providers. All Dutch healthcare professionals should provide palliative care, and for complicated cases, palliative care expert teams can be consulted.

The study was performed at the UMCG, an academic hospital, and one of fourteen melanoma treatment centers in the Netherlands. Approximately 40 new patients with melanoma brain metastases are treated yearly. For these patients, all registered, standard of care, localized and systemic treatments are available. A dedicated team, including medical oncologists, neurologists, radiation oncologists, neurosurgeons, pathologists, and radiologists, are involved in the care. Furthermore, the UMCG has a palliative care expert team that can become involved in patient’s care upon request by the treating physician.

### Study characteristics

Retrospective hospital chart review was performed. Patient (age, gender) and tumor (*BRAF-*mutational status, LDH-level, interval between metastatic melanoma diagnosis and brain metastases, number of brain metastases, presence of extracranial metastases, disease status 3 months before death, and time between brain metastases diagnosis and death) characteristics were extracted. If patients were diagnosed with brain metastases in the last 3 months of life, the disease status was assessed at time of brain metastases diagnosis. The last 3 months of life were extensively examined, including received anti-tumor treatment, healthcare consumption, presence of neurological symptoms, presence of clinical or radiological intracranial progression at the last hospital visit, and documentation of a do-not-resuscitate (DNR) status. Hospital healthcare consumption included: outpatient clinical appointments, medical imaging appointments, ER visits, and hospitalizations. For the received treatments in the last 3 months of life, we determined the last day of systemic therapy as the day of the last infusion for intravenous therapy, and for BRAF/MEK-inhibitors, it was the day patients refilled their last prescription. BRAF/MEK-inhibitors are usually prescribed for one month in the UMCG. The occurrence of BRAF/MEK-inhibition post-progression and re-challenge with BRAF/MEK-inhibition were also determined. Post-progression BRAF/MEK-inhibition was defined as the continuation of BRAF/MEK-inhibitors after radiological or clinical progression. A re-challenge with BRAF/MEK-inhibitors was defined as retreatment with BRAF/MEK-inhibitors with at least one month between discontinuation and retreatment. To determine the available treatment lines in the last 3 months of life, we also extracted the received systemic treatments prior to the last 3 months of life. The initial treatment goal 3 months before death was retrospectively determined. Patients were divided into two groups according to the treatment goal: patients being treated with the aim of long-term survival and patients being treated with palliative intent. Post-progression and re-challenge BRAF/MEK-inhibitor, chemotherapy, provided treatments in patients with WHO-performance status of at least two, and receiving best supportive care only were all considered as treatments with palliative intent.

### Statistical analyses

Statistical analyses were performed using SPSS Version 24.0 (IBM SPSS Statistics, Armonk, NY). The outcomes were described using mean (standard deviation) and median (range) for parametric and non-parametric continuous variables, respectively. Categorical variables were presented in numbers and percentages. Data distribution was examined using Kolmogorov–Smirnov tests, histograms, and Q-Q plots. Differences in the number of patients that received systemic and localized treatment near death between patients with or without a *BRAF-*mutation were examined using chi-square tests. The differences in the number of patients receiving systemic treatments in the last 3 months of life between patients that had or had not received all available classes of systemic treatments, between patients with different treatment goals 3 months before death, and between disease status (intracranial, intra- and extracranial, or no progression) 3 months before death were also explored using chi-square tests. “All available classes of systemic treatments” was defined as having received both immune checkpoint inhibitors (anti-PD-1 and/or anti-CLTA-4) and BRAF/MEK-inhibitors for patients with a *BRAF-*mutation and immune checkpoint inhibitors in patients without a *BRAF-*mutation. Furthermore, also using chi-square tests, the number of patients visiting the ER or being hospitalized were compared between patients with and without systemic treatment during the last 3 months of life, between patients with different treatment goals, and between disease status (intracranial, intra- and extracranial, or no progression) 3 months before death. Fisher’s exact tests were performed instead of chi-square tests in case of too small subgroups. The differences in the number of days in the hospital, days being admitted, and interval between last hospital visit and death in patients with and without systemic treatment were determined using Mann–Whitney U tests.

## Results

### Patient characteristics

Between June-2015 and June-2018, 140 patients were diagnosed with melanoma brain metastases, of which 100 died before November-2019 and were included. Age at time of brain metastases diagnosis was 65 years (median, range: 27–90), 57 patients (57%) were male, and a *BRAF-*mutation was present in 66 patients (66%, Table 1). In over half of the patients, the brain metastases were diagnosed at the time of initial metastatic melanoma diagnosis (59%, *n* = 59), and in 17 patients (17%) the brain metastases were diagnosed at least 1 year after the diagnosis of metastatic melanoma. The median interval between the diagnosis of brain metastases and death was 5.7 months (range: 0–42), and 29 patients (29%) died within 3 months after brain metastases diagnosis. Twenty-nine patients (29%) had intracranial progression only, 35 patients (35%) had intra- and extracranial progression, and 34 patients (34%) had no disease progression all 3 months before death (Table 1).

**Table 1:** Clinical and disease characteristics

	**N (%)** ***N* = 100**
**Median age,** years (range) ^a^	65 (27–90)
**Male**	57 (57%)
***BRAF*-mutation present**	66 (66%)
**LDH **^**a**^	
< 1 ULN	57 (57%)
1–2.5 ULN	27 (27%)
> 2.5 ULN	9 (9%)
Missing	7 (7%)
**Time between metastatic melanoma diagnosis and brain metastases**	
0 months	59 (59%)
1 – 6 months	16 (16%)
6 – 12 months	8 (8%)
> 12 months	17 (17%)
**Symptomatic brain metastases **^**a**^	68 (68%)
**Number of brain metastases **^**a**^	
1	28 (28%)
2–5	28 (28%)
> 5	37 (37%)
Leptomeningeal disease	7 (7%)
**Interval between diagnosis brain metastases and death,** months (range)	5.7 (0–42)
**Disease status **^**b**^	
Only intracranial progression	29 (29%)
Only extracranial progression	2 (2%)
Both	35 (35%)
None	34 (34%)
**Received systemic treatments before the last 3 months of life** **Patients with a *BRAF*-mutation (*n* = 66)**	
ICI	2 (3%)
BRAF/MEK-inhibitors	23 (35%)
ICI and BRAF/MEK-inhibitors	31 (47%)
Chemotherapy	3 (5%)
**Patients without a *BRAF-*mutation (*n* = 34)**	
ICI	13 (38%)
Chemotherapy	2 (6%)

*Abbreviations*: *ULN* upper limit of normal, *ICI* immune checkpoint inhibitors

^a^At time of brain metastases diagnosis, ^b^At 3 months prior to death

### Anti-tumor treatments

**Fig. 1:**
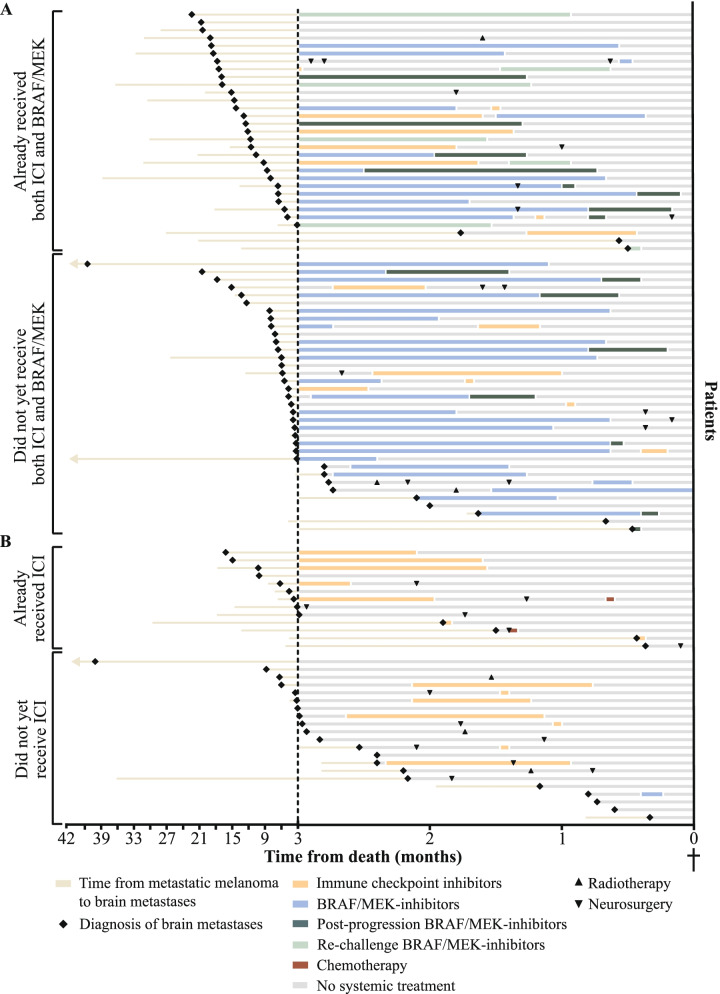
Swimmer plot of time between metastatic melanoma and death, including treatments received for melanoma brain metastases in last 3 months of life. Patients are stratified by *BRAF*-mutational status and treatments received prior to the last 3 months of life **A**: patients with a *BRAF-*mutation, **B**: patients without a *BRAF-*mutation

After diagnosis of metastatic melanoma, 94 patients (94%) received systemic and/or localized (WBRT, SRT, or (neuro)surgery) treatment. Before the last 3 months of life, 31 out of 66 patients (47%) with a *BRAF*-mutation had already received both immune checkpoint inhibitors and BRAF/MEK-inhibitors, and 13 out of 34 patients (38%) without a *BRAF*-mutation received immune checkpoint inhibitors. During the last 3 months of life, 83 patients (83%) received anti-tumor treatment. Fifty-seven patients (57%) were treated with the aim to induce long-term disease control, and 43 patients (43%) received treatment with palliative intent. Figure 1 shows a swimmer plot of treatments received in the last 3 months of life categorized by the presence of a *BRAF*-mutation and having received immune checkpoint inhibitors and BRAF/MEK-inhibitors, in case a *BRAF*-mutation is present, before the last 3 months of life.

During the last 3 months of life, 72 patients (72%) received systemic anti-tumor treatment, 34 patients (34%) in the last month, and six (6%) in the last week of life. Patients with a *BRAF-*mutation were more likely to receive systemic treatment during the last 3 months (85% vs. 47%, *p* < 0.001) and the last month of life (42% vs. 18%, *p* = 0.01) than patients without a *BRAF-*mutation (Table 2). Of the 66 patients with a *BRAF-*mutation, 49 patients (74%) received BRAF/MEK-inhibition in the last 3 months of life. BRAF/MEK-inhibition was continued post-progression in sixteen patients, and seven patients were re-challenged with BRAF/MEK-inhibition in the last 3 months. The number of patients receiving systemic anti-tumor treatment in the last 3 months (77% vs. 68%, *p* = 0.30) and last month (34% vs. 34%, *p* = 0.59) and last week (5% vs. 7%, *p* = 0.59) of life did not significantly differ between patients that had already received all available classes of systemic treatments prior to the last 3 months of life compared to patients that did not receive those treatments. The number of patients that received systemic treatment in the last 3 months, last month, and last week of life did not differ between treatment goals (data not shown). A difference in the number of patients receiving systemic treatment in the last 3 months of life was observed between patients with intracranial progression only, intra- and extracranial progression, and patients without disease progression 3 months before death (69% vs. 57% vs. 88%, *p* = 0.02). No differences were observed in number of patients receiving systemic treatment in the last month or week of life.

**Table 2:** Number of patients receiving anti-tumor treatment near death

	**Overall** ***N* = 100** **n (%)**	**BRAF + ** ***N* = 66** **n (%)**	**BRAF -** ***N* = 34** **n (%)**	***P*-value** **X**^**2**^
**Systemic treatment**				
last 3 months Last month Last week	72 (72%) 34 (34%) 6 (6%)	56 (85%) 28 (42%) 5 (8%)	16 (47%) 6 (18%) 1 (3%)	< 0.001 0.01 0.36
**Localized treatment**				
last 3 months Last month Last week	40 (40%) 14 (14%) 4 (4%)	22 (33%) 9 (14%) 3 (5%)	18 (52%) 5 (15%) 1 (3%)	0.06 0.88 0.58 ^a^

^a^Fisher’s exact test

In the last 3 months of life, 40 patients (40%) received localized treatment. During this period, fewer patients with a *BRAF-*mutation received localized treatment than patients without a *BRAF-*mutation, however, this difference was not significant (33% vs. 52%, *p* = 0.06, Table 2). No differences between patients with and without a *BRAF-*mutation were found in the number of patients receiving localized treatment in the last month or last week of life.

In the last 3 months of life, 26 patients (26%) received cranial irradiation (11 SRT, 15 WBRT), nine patients (9%, 7 WBRT, 2 SRT) in the last month, and 3 (3%, 3 WBRT) in the last week of life. The indications for cranial irradiation in the last month of life were neurological symptoms (*n* = 7), progressive asymptomatic brain metastases (*n* = 2), and postoperative radiotherapy (*n* = 1). Twelve patients (12%) received extracranial radiation in the last 3 months of life, and six patients (6%) in the last month, one patient (1%) in the last week. Indications for extracranial radiation in the last month of life were painful boney metastases (*n* = 3), spinal cord compression (*n* = 2), and symptomatic lymph node metastasis (*n* = 1). Four out of fourteen patients who received radiotherapy in the last month of life could not complete their planned radiotherapy regime (3 WBRT and 1 radiotherapy for spinal cord compression). In three of these patients, this was due to clinical deterioration, and one patient expressed wishes for euthanasia due to uncontrollable pain.

In the last 3 months of life, six (6%) patients underwent a neurosurgical procedure. The indications were neurological symptoms (*n* = 2), large brain metastasis likely to cause symptoms soon (*n* = 1), and the need for pathological tumor tissue analysis (*n* = 3).

Reasons for cessation of treatment or not to commence treatment were poor performance status (*n* = 58, 58%), lacking therapeutic options (*n* = 18, 18%), patient’s preferences (*n* = 7, 7%), and treatment complications (*n* = 1, 1%). One patient who received BRAF/MEK-inhibitors died between two outpatient clinic visits, and it was unclear if the anti-tumor treatment was stopped prior to death. BRAF/MEK-inhibition was continued in 16 patients (16%) at a time when the majority of care was already transferred to the GP, this included patients receiving post-progression and re-challenge BRAF/MEK-inhibition. BRAF/MEK-inhibitors were stopped in those patients when the clinical situation deteriorated further, in close collaboration between the GP and the oncologist.

### Healthcare consumption

Patients were at the hospital on a median of nine separate days during the last 3 months of life (range: 0–38). This included all outpatient clinic and medical appointments, ER visits, and hospitalizations. In the 97 patients who visited the hospital during the last 3 months of life, the median interval between the last hospital visit and death was 18 days (range: 0–88). The interval between last visit and death was significantly shorter in patients who received systemic treatment near death than those who did not (15 vs. 38 days, *p* = 0.003, Table 3).

**Table 3:** Healthcare consumption in the last 3 months of life

	**Overall** ***N* = 100**	**Systemic treatment in last 3 months** ***N* = 72**	**No systemic treatment in last 3 months** ***N* = 28**	***P*-value** **Mann–Whitney U** **or X**^**2**^
**Number of days in hospital,** median (range)	9 (0–38)	11 (1–38)	4 (0–30)	0.002
**Patients visiting the ER,** n (%)	64 (64%)	54 (75%)	10 (36%)	< 0.001
**Patients being hospitalized,** n (%)	63 (63%)	54 (75%)	10 (36%)	< 0.001
**Number of days admitted,** median (range)	3 (0–34)	4 (0–34)	0 (0–25)	0.008
**Days between last hospital visit and death,** median (range) ^a^	18 (0–88)	15 (0–81)	38 (0–88)	0.003

^a^In patients that visited the hospital in the last 3 months of life (*n* = 9)

During the last 3 months of life, 64 patients (64%) visited the ER, with a total of 107 visits. Of the 107 visits, 68 (64%) were related to neurological symptoms, and the main complaints were neurological deficits (*n* = 22), impaired consciousness (*n* = 13), headaches (*n* = 12), nausea and vomiting (*n* = 11), and seizures (*n* = 10). Only a small proportion of visits were likely related to anti-tumor treatment (*n* = 9, 8%), including fever, cerebral edema after radiotherapy, and surgical wound infections. Patients receiving systemic treatment during the last 3 months of life were significantly more likely to visit the ER than patients not receiving systemic treatment (75% vs. 36%, *p* < 0.001, Table 3). More patients that were treated with the aim of long-term survival visited the ER compared to patients that received treatment with palliative intent (74% vs. 51%, *p* = 0.02). The number of patients visiting the ER differed with a trend to significance between patients with intracranial, intra- and extracranial, and no disease progression 3 months before death (62% vs. 51% vs. 79%, *p* = 0.05).

In the last 3 months of life, 63 patients (63%) were hospitalized, with a total of 100 hospitalizations. Of these 100 hospitalizations, 62 (62%) were related to brain metastases. Reasons for hospitalization included, among others, focal motor deficits (*n* = 12), nausea and vomiting (*n* = 9), seizures (*n* = 9), and headache (*n* = 9). Nine patients (9%) died while being hospitalized. Six of these deaths were due to brain metastases. Significant differences in the number of patients (75% vs. 36%, *p* < 0.001) and the number of days (4 vs. 0 days, *p* = 0.008) being admitted in the last 3 months of life were observed between patients that received systemic treatment in the last 3 months of life than those that did not (Table 3). More patients that were treated with the aim of long-term survival were hospitalized during the last 3 months of life compared to patients that received treatment with palliative intent (78% vs. 47%, *p* = 0.002). The number of patients being hospitalized did not significantly differ according to the presence of intracranial, intra- and extracranial, and no progression 3 months before death (62% vs. 57% vs. 71%, *p* = 0.51).

### Presence of neurological symptoms

At the time of brain metastases diagnosis, 68 patients (68%) experienced neurological symptoms, and 92 patients (92%) experienced symptoms in the last 3 months of life. Those symptoms included, among others, headache (*n* = 38), motor deficits (*n* = 37), cognitive impairment (*n* = 37), epilepsy (*n* = 34), speech deficits (*n* = 30), vomiting (*n* = 28), and impaired consciousness (*n* = 28).

### Resuscitation status

Evidence for a DNR was found in 56 patients (56%). In 37 of those patients (66%), the DNR was documented in the electronic charts within the last 3 months, for seventeen in the last month (30%), and for four in the last week of life (7%).

## Discussion

This study provides insight into the anti-tumor treatment and hospital healthcare consumption, near death, of patients with melanoma brain metastases in the current treatment era. The majority of patients received anti-tumor treatment during the last 3 months of life. Having a *BRAF-*mutation was associated with more patients receiving anti-tumor treatment in the last 3 months. Receiving systemic treatment within the last 3 months of life was associated with an increased likelihood of visiting the ER and being hospitalized.

Compared to our study, reported an older study a lower number of patients receiving systemic treatment near death. This study reported 20% of patients that died due to melanoma in Massachusetts and California (USA) in 1996 receiving chemotherapy during the last 3 months of life [38]. Data from the National Institute’s Surveillance, Epidemiology and End Results Medicare Database showed that between 2000 to 2007, 17% of metastatic melanoma patients aged ≥ 65 received chemotherapy during the last 3 months of life [33]. Fifty-two percent of patients with metastatic melanoma who died in France’s hospitals between 2010 to 2013 received systemic treatment during the last 3 months of life and 26% in the last month [32]. The latter study differs from this study, as they only included patients that died while being hospitalized. Furthermore, most of the previous studies were performed before the implementation of immune checkpoint inhibitors and targeted therapies that can, unlike chemotherapy, induce intracranial responses. The availability of effective treatment options resulted in a higher percentage of patients with melanoma brain metastases receiving systemic treatment near death in our study compared to older studies. For the development of future care pathways for patients with melanoma brain metastases, it is essential to acknowledge the increased use of systemic treatment near death and the need for a strategy that focuses on simultaneously providing anti-tumor treatments and palliative care in these patients.

BRAF/MEK-inhibitors provide a treatment option with rapid onset of response and can, thereby, be used to minimize symptom burden in the last phase of life [20, 21]. In the current study, most patients with a *BRAF-*mutation received BRAF/MEK-inhibitors during the last 3 months of life, including patients who received post-progression treatment or were re-challenged with BRAF/MEK-inhibitors. Rapid intracranial progression after discontinuation of BRAF/MEK-inhibitors and efficacy of re-challenge BRAF/MEK-inhibitors has been reported [24–27], and this knowledge may prompt BRAF/MEK-inhibition near death. In the present study, some patients continued BRAF/MEK-inhibition while most of their care had been transferred to the GP. This requires intensive collaboration between medical oncologists and GP’s near the death of the patient. This situation may also arise in patients with other tumor types that are treated with targeted therapies. Therefore, future research should focus on the impact of post-progression treatment and re-challenge with targeted therapies on symptom burden and patients, relatives, and GP’s perception on the continuation of targeted therapies near death.

The observed number of patients (27%) receiving radiotherapy during the last months of life was relatively high. Earlier studies in cancer patients with and without brain metastases reported 5 to 11% of patients receiving radiotherapy in the last 3 months of life [32, 40–42]. In the last decade, SRT has been increasingly used to achieve intracranial tumor control and avoid neurological symptoms while waiting to respond to immune checkpoint inhibitors [9, 43, 44]. The higher incidence of radiotherapy may be explained by the combination of the increased usage of SRT and radiotherapy for palliation. The latter is known to be commonly provided near death [41, 45].

Over 60% of patients visited the ER and/or were admitted to the hospital near death. In 2017 in the Netherlands, 59% of cancer patients were admitted to the hospital in the last 3 months of life, and 27% visited the ER in the last month of life [46, 47]. Other studies, not limited to patients with brain metastases, reported 30 to 74% of patients visiting the ER and 41 to 55% being admitted in the last 3 months of life [33, 48–50]. We observed that neurological symptoms were most often the reason for ER visits and hospitalization. The high symptom burden associated with brain metastases may explain the high number of patients visiting the ER and being admitted in this cohort.

Receiving systemic treatment near death was associated with more patients visiting the ER and being hospitalized in the last 3 months of life. However, fewer patients treated systemically with palliative intent and those receiving best supportive care visited the ER and were hospitalized than patients still treated with the aim of long-term survival. ER visits and hospitalizations may be unwanted in the last phase of life, especially since most patients prefer to remain at home. Advance care planning, including conversations about medical decisions and interventions to deal with symptoms, can avoid ER visits and hospitalizations near death [51]. Furthermore, providing the GP’s and community nurses with guidance on how to control complications of melanoma brain metastases in a home setting can also help improve care at home and minimize hospital healthcare consumption near death. Information for the GP’s on rescue therapies for seizures, continuation of anti-epileptic drugs in patients with a history of seizures and dysphagia, and the usage of corticosteroids to alleviate vasogenic edema-induced symptoms can help the GP’s to tackle common brain metastases symptoms in a home setting.

Most patients had clinical or radiological intracranial progression at the last hospital visit, potentially indicating that these patients died of a neurological cause. In the Netherlands, the care for terminally ill patients is often transferred to the GP and healthcare at home is available for those in need. This end-of-life care strategy aims to facilitate patients to die at home, which is the most often preferred place of death. In concordance with this strategy, only a few patients in our study died in the hospital. The retrospective design of the study together with the low number of patients dying in the hospital limited us determining the exact cause of death in our cohort and warrants attention in further prospective studies.

The study’s main limitations are the relatively small sample size, single-center (academic hospital), and retrospective nature. Treatment decisions are made in real-time, and the evaluation of treatment decisions retrospectively is artificial. The disease trajectory of patients with melanoma brain metastases is uncertain, and identification of the end-of-life phase is difficult. Factors such as patient’s preferences, performance status, and clinical characteristics may influence treatment decisions. Unfortunately, these factors are not routinely documented extensively, and thus, we were unable to explore the impact of these factors in our study. Longitudinal studies following patients from diagnosis to death or long-term survival could provide additional information regarding treatment decision-making. To provide additional insight into end-of-life care of patients with melanoma brain metastases, future studies also need to prospectively examine the impact of ongoing treatment near death on quality of life (including impact of treatment toxicity experienced), care satisfaction, and healthcare costs (e.g., costs per quality-adjusted life year). We included patients diagnosed with brain metastases between June-2015 and June-2018, and that had died before November-2019. Therefore, patients with longer survival times were not included in the current study. Nevertheless, it is uncertain whether the last 3 months of life of patients with short survival differ from those with longer survival. Lastly, we only evaluated the healthcare consumption of the patients in our hospital. Therefore, we might have missed healthcare consumptions at other hospitals. Since melanoma care is centered to fourteen hospitals in the Netherlands and patients contacting their treating hospital in case of problems, we expect that the healthcare consumption at other hospitals is minimal. Furthermore, since palliative care in the Netherlands is primarily provided by the treating physician and the GP, we were unable to examine the effect of referring patients specifically to Palliative Medicine on healthcare consumption near death.

## Conclusions

The majority of patients received anti-tumor treatment in the last 3 months of life. In the management of melanoma brain metastases, clinicians face complex discussions with their patients about prognosis and treatment, including the possibilities of long-term survival or rapid deterioration and death. The study results provide insight into the current end-of-life care of patients with melanoma brain metastases in terms of anti-tumor treatment and healthcare consumption. This may provide a basis for the development of care pathways, with integrated palliative care, for patients with melanoma brain metastases in the current treatment era and guide further research on the impact of ongoing treatment near death on quality of life (including symptom burden and treatment toxicity) and care satisfaction.

## Data Availability

The dataset generated and analyzed used in the current study is available from the corresponding author on reasonable request.

## Abbreviations

BRAF: V-raf murine sarcoma viral oncogene homolog B1
DNR: Do-not-resuscitate
ER: Emergency department
GP: General practitioner
LDH: Lactate dehydrogenase
MEK: Mitogen-activated extracellular signal-regulated kinase
SRT: Stereotactic radiotherapy
UMCG: University Medical Center Groningen
ULN: Upper limit of normal
WBRT: Whole-brain radiation
